# 

**Published:** 1888-11

**Authors:** 


PROFESSOR ELKANAH WILLIAMS, M. D.

### ANNOUNCEMENTS.

The sixteenth annual meeting of the American Public Health Association, will be held in Milwaukee, Wisconsin, Tuesday, Wednesday, Thursday, and Friday, November 20, 21, 22, 23, 1888.

The next annual meeting of the American Academy of Medicine, will be held in New York, on November 13 and 14, 1888.

Volume LVII.] NOVEMBER, 1888. [Number 5.

### The CHICAGO MEDICAL JOURNAL AND EXAMINER

EDITOR:
S. J. JONES, M.D., LL.D.

COLLABORATORS:
CHICAGO:

Professor E. ANDREWS, M.D., LL.D.
Professor J. BARTLETT, M.D.
Professor W. T. BELFIELD, M.D.
Professor F. BILLINGS, M.D.
Professor W. H. BYFORD, A.M., M.D.
Professor L. CURTIS, A.M., M.D.
Professor N. S. DAVIS, Jr., A.M., M.D.
Professor O. C. DE WOLF, A.M., M.D.
Professor C. FENGER, M.D.
Professor J. N. HYDE, A.M., M.D.
Professor E. F. INGALS, A.M., M.D.
Professor F. S. JOHNSON, A.M., M.D.
Professor H. A. JOHNSON, M.D., LL.D.
Professor C. W. PURDY, M.D.
Professor C. G. SMITH, A.M., M.D.
Professor E. WING, A.M., M.D.
Professor E. C. DUDLEY, A.B., M.D.

MILWAUKEE, WIS.:
Professor N. SENN, M.D., Ph.D.

WASHINGTON, D. C.:
S. O. RICHEY, M. D.

UNITED STATES ARMY:
Surgeon D. L. HUNTINGTON, M.D.

UNITED STATES NAVY:
Surgeon-General J. M. BROWNE, M.D. Medical-Director A. L. GIHON, A.M., M.D.

MARINE HOSPITAL SERVICE:
Surgeon S. T. ARMSTRONG, M.D., Ph.D.

